# Colibactin: More Than a New Bacterial Toxin

**DOI:** 10.3390/toxins10040151

**Published:** 2018-04-10

**Authors:** Tiphanie Faïs, Julien Delmas, Nicolas Barnich, Richard Bonnet, Guillaume Dalmasso

**Affiliations:** 1 Université Clermont Auvergne, Inserm U1071, M2iSH, USC-INRA 2018, F-63000 Clermont-Ferrand, France; tfais@chu-clermontferrand.fr (T.F.); jdelmas@chu-clermontferrand.fr (J.D.); nicolas.barnich@uca.fr (N.B.); guillaume.dalmasso@uca.fr (G.D.); 2 CHU Clermont-Ferrand, Laboratoire de Bactériologie, Centre de Biologie, F-63003 Clermont-Ferrand, France

**Keywords:** colibactin, *pks*, *E. coli*, cancer, toxin, microbiota

## Abstract

Cyclomodulins are bacterial toxins that interfere with the eukaryotic cell cycle. A new cyclomodulin called colibactin, which is synthetized by the *pks* genomic island, was discovered in 2006. Despite many efforts, colibactin has not yet been purified, and its structure remains elusive. Interestingly, the *pks* island is found in members of the family *Enterobacteriaceae* (mainly *Escherichia coli* and *Klebsiella pneumoniae*) isolated from different origins, including from intestinal microbiota, septicaemia, newborn meningitis, and urinary tract infections. Colibactin-producing bacteria induce chromosomal instability and DNA damage in eukaryotic cells, which leads to senescence of epithelial cells and apoptosis of immune cells. The *pks* island is mainly observed in B2 phylogroup *E. coli* strains, which include extra-intestinal pathogenic *E. coli* strains, and *pks* *E. coli* are over-represented in biopsies isolated from colorectal cancer. In addition, *pks* *E. coli* bacteria increase the number of tumours in diverse colorectal cancer mouse models. Thus, colibactin could have a major impact on human health. In the present review, we will focus on the biological effects of colibactin, the distribution of the *pks* island, and summarize what is currently known about its synthesis and its structure.

## 1. Introduction

Cyclomodulins are bacterial toxins that interfere with the eukaryotic cell cycle. Until 2006, three types of cyclomodulins were known in *Escherichia coli*: two able to inhibit proliferation (cytolethal distending toxin, CDT; and the cycle inhibiting factor, Cif) and one able to promote proliferation (cytotoxic necrotizing factor, CNF) [1]. In 2006, Nougayrède and colleagues identified in the *E. coli* meningitis strain IHE3034 a toxin that they named colibactin [2]. Colibactin is a natural and genotoxic chemical compound which is synthetized by polyketide synthases, non-ribosomal peptide synthases, and hybrid enzymes encoded by a 54-kb genomic island designated *pks* [2]. This toxin induces DNA double-strand breaking, chromosome aberrations, and cell cycle arrest in the G2/M phase [2,3]. Interestingly, *pks*-harbouring *E. coli* (*pks E. coli*) have been isolated from intestinal microbiota as commensal bacteria [4,5,6,7,8], and in infectious diseases such as septicaemia [5,9], newborn meningitis [10], and urinary tract infections [4,11]. In addition, colibactin-producing *E. coli* are over-represented in colorectal cancer (CRC) [12,13,14,15] and they increase the number of tumours in various CRC mouse models [14,16,17,18]. This toxin could therefore have a significant effect on human health.

It is known that *pks*-related islands produce numerous compounds [19]. Hence, activities other than genotoxicity have been researched. Biological studies have clearly shown that colibactin-producing bacteria possess anti-inflammatory [20], antibiotic [21,22] and analgesic effects [23]. Thus, instead of producing one compound, the *pks* island probably produces several, in which case it would be interesting to purify them to investigate their bioactivities. However, despite many efforts, the structure of these compounds still remains partially elusive.

Here we will give an overview of what is currently known about the epidemiology of the *pks* island, and about the synthesis, predictive structure, and biological activities of colibactin.

## 2. Distribution of the *pks* Island

The *pks* island was initially identified in the *E. coli* strain IHE3034 isolated from neonatal bacterial meningitis [2]. Epidemiological studies have shown that *pks* can also be found in the species *Klebsiella pneumoniae*, *Enterobacter aerogenes*, and *Citrobacter koseri*.

### 2.1. Escherichia coli

*E. coli* which harbour *pks* are relatively frequent and can be isolated from multiple sites of the human body. Independent studies have reported the presence of *pks E. coli* in the gut microbiota, with a prevalence in stools ranging from 12% to 32% [4,5,6]. A study conducted on 130 Swedish newborns showed that in their faeces 33% of *E. coli* tested carried the *pks* island [7]. These results were confirmed by a study performed in France on 184 healthy neonates in whom 26.9% of *E. coli* isolated from faecal samples were *pks* positive [8]. *E. coli* is one of the main causes of urinary tract infections [24]. Dubois et al. screened 146 urosepsis *E. coli* strains and found that 32% of them were positive for *pks* [4]. Another study, involving 18 *E. coli* strains responsible for prostatitis, showed that 72% harboured *pks* [11]. *E. coli* strains expressing the K1 capsule are a major cause of sepsis and of newborn meningitis [25]. The *pks* island has also been reported to be strongly associated with *E. coli* K1: of 34 isolates examined, *pks* was found in 33 [10]. Finally, 31.5–58% of *E. coli* found in blood cultures also tested positive for *pks* [5,9].

*E. coli* are divided into eight phylogenetic lineages, A, B1, B2, C, D, E, F, and clade I [26]. Almost all *E. coli* belong to the phylogroups A, B1, B2, and D and classically the extra-intestinal pathogenic *E. coli* (ExPEC) strains belong to group B2 [27]. Interestingly, the *pks* island is strongly associated with *E. coli* strains of phylogroup B2 [5,6,7,12,28]. Strains isolated in countries with a westernized life-style belong mainly to the B2 group and their prevalence is constantly increasing [29]. Thus, it is tempting to speculate that in the future the prevalence of *pks E. coli* will also rise. Interestingly, the *pks* island is frequently associated with other virulence factors such as other cyclomodulins, adhesins, or ExPEC-associated virulence genes (adhesins, haemolysins, toxins, siderophores) [4,5,6,12,15]. Thus, it seems that the *pks* island is associated with a particularly high virulent subgroup of B2 strains.

### 2.2. Klebsiella pneumoniae

The first report of the presence of *pks* in *K. pneumoniae* was published by Putze et al. in 2009. Of the 141 isolates screened, 5 (3.5%) were positive for *pks* [6]. In 2014, Lai and colleagues observed the presence of the island in 25.6% of the 207 *K. pneumoniae* strains they tested [30]. The discrepancy between these studies could be explained by the origin of the strains tested. The strains studied by Putze et al. came from a European collection while those studied by Lai et al.*,* came from a collection in Taiwan. The highly virulent serotype K1 is the most frequent in Taiwan [31], but is ranked only eighth in Europe [32], and most K1 strains (66%) in the study conducted by Lai et al. appeared to be positive for *pks* [30]. The overrepresentation of *pks* in K1 strains was confirmed by screening a total of 400 *K. pneumoniae* isolates showing that 78.8% of K1 strains were *pks* positive [33].

### 2.3. Other Members of the Family Enterobacteriaceae: Enterobacter aerogenes and Citrobacter koseri

To date, only the study performed by Putze et al. has investigated the presence of *pks* in *Enterobacter aerogenes* and *Citrobacter koseri* [6]. 27.3% of *E. aerogenes* (*n* = 3/11) and 100% of *C. koseri* isolates (*n* = 1/1) harboured *pks*. However, owing to the small number of isolates tested, further studies are needed to confirm and establish the prevalence of *pks* in these species.

## 3. Colibactin Biosynthesis

**Figure 1:**
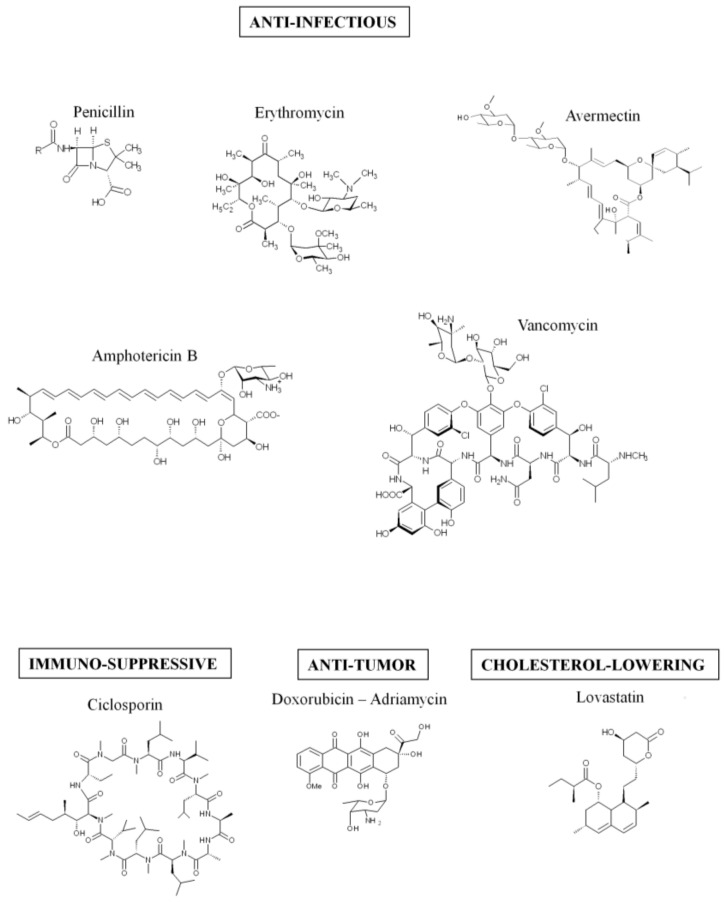
Example of molecules synthetized by the hybrid non-ribosomal peptide synthetase-polyketide synthase (NRPS-PKS) assembly lines.

**Figure 2:**
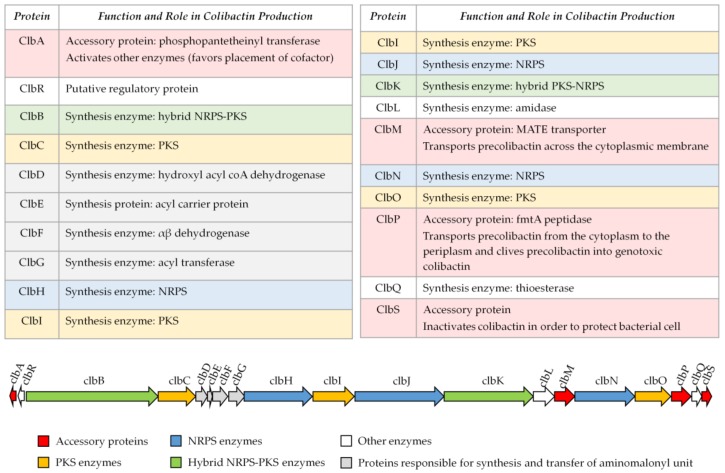
Organization of the *pks* island. Adapted from Reference [2].

Colibactin is synthesized by a hybrid non-ribosomal peptide synthetase-polyketide synthase (NRPS-PKS) assembly line [2] and is a member of a large family of molecules with multiple functions. Some of them are commonly used in human health, for example, to treat bacterial infections (the antibiotics erythromycin, tetracyclin, penicillin), cancers (the chemotherapeutic drug bleomycin), and graft rejection (the immunosuppressive drug rapamycin) [19] (Figure 1). The assembly line responsible for colibactin synthesis is encoded within the 54-kilobase genomic island *pks* representing a total of 19 genes (*clbA* to *clbS*) (Figure 2) [2]. This machinery consists of three non-ribosomal peptide megasynthases (NRPS: ClbH, ClbJ, ClbN), three polyketide megasynthases (PKS: ClbC, ClbI, ClbO), two hybrid NRPS/PKS megasynthases (ClbB, ClbK), and nine accessory, tailoring and editing enzymes. When the *pks* island was discovered, by Nougayrède and colleagues, they performed a systematic mutagenesis of the *pks*-island genes and found that all of the PKS and NRPS proteins and eight of the nine accessory and tailoring enzymes were required to induce the cytopathic effect mediated by *pks E. coli* [2]. NRPS, PKS, and hybrid NRPS-PKS are usually organized in mega-complexes as an assembly line, in which the synthesized compound is transferred from one enzymatic module to the following one. Colibactin belongs to a subset of hybrid polyketide-non-ribosomal peptides that undergo a prodrug activation mechanism which involves installation of a structural motif at the N-terminus, which is removed in the final stage of biosynthesis [34,35].

At the beginning of the assembly line, ClbA, a phosphopantetheinyl (PPant) transferase, is responsible for the activation of the NRPS and PKS enzymes by the addition of a PPant onto the NRPS and PKS carrier protein domains. Interestingly, ClbA is also involved in the synthesis of the siderophores enterobactin and yersiniabactin by replacing EntD. Hence, there is evidence of a possible trans-complementation between PPants in *E. coli* and therefore a connection between siderophore and colibactin production [36]. The enzymatic modules are then loaded with a building block according to their domain architecture: acetyl-, malonyl-, or methylmalonyl-CoA monomers for PKS or amino acid monomers; and proteinogenic or non-proteinogenic amino acids for NRPS. These building blocks are sequentially incorporated into the synthetized compound during the progression along the colibactin-assembly line and modified by the editing enzymes.

**Figure 3:**
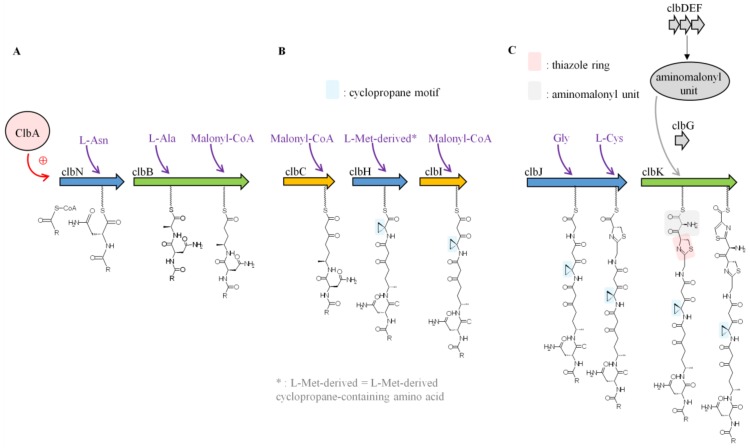
Schematic representation of colibactin biosynthesis. Adapted from Reference [39]. (**A**) First steps of colibactin synthesis. (**B**) Cyclopropane formation. (**C**) Thiazole ring formation.

The first study aimed at elucidating colibactin biosynthesis focused on ClbN and ClbB, the NRPS enzymes involved in the initiation of colibactin biosynthesis [35]. It was found that ClbN uses asparagine to generate *N*-myristoyl-d-Asn, a prodrug motif, which is accepted by ClbB [35]. ClbB adds an L-amino acid (Ala or Val) and then incorporates a malonyl-CoA into the intermediate [35] (Figure 3A). Synthesis of precolibactin continues with the NRPS-PKS assembly line (ClbC–H–I–J–K) using as substrates malonyl-CoA and amino acids including Gly, Cys, and the L-Met-derived cyclopropane-containing amino acid [37,38]. ClbH and ClbI are essential for cyclopropane (C_3_H_3_) formation [38,39] (Figure 3B), which is responsible for colibactin-induced DNA alkylation [40]. Recent in vitro enzymatic studies have shown that the ClbD–G enzymes are responsible for the synthesis and attachment of the unusual PKS extender aminomalonyl unit (AM) [37,41]. Mutants of *pks E. coli* which lack any component of the AM biosynthetic machinery are not genotoxic [2], suggesting that these enzymes and therefore AM incorporation are critical for constructing genotoxic metabolites. ClbG recognizes AM and transfers it to ClbK, which incorporates AM into colibactin [37] (Figure 3C). Interestingly, ClbG could transfer AM to multiple enzymes of the *pks* assembly line (ClbC, ClbK, ClbO, ClbI) suggesting that AM could be incorporated more than once into colibactin [37,42]. Another important structure suspected to be important for colibactin biological function are thiazole rings, which are heterocyclic structures containing both sulphur and nitrogen (C_3_H_3_NS) generated by ClbK [39] (Figure 3C). PKS enzyme ClbO could be the final enzymatic module of the colibactin-assembly line. However, colibactin synthesis can generate multiple precolibactin intermediates or derailment products [42,43].

**Figure 4:**
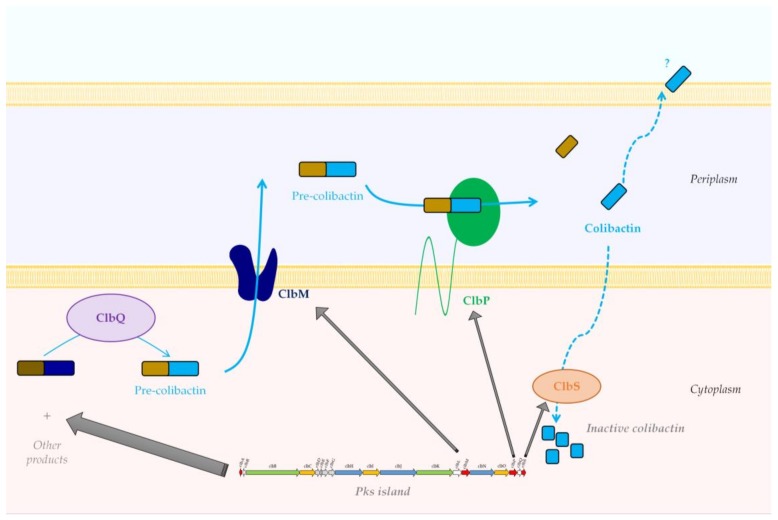
Proposed model for final maturation of colibactin. ClbM takes charge of the prodrug, and releases it into the periplasm, where ClbP generates by cleavage the mature colibactin. ClbS is able to sequester and inactivate any colibactin presents in the cytoplasm, therefore protecting bacterial DNA.

The off-loading mechanism of compounds from the colibactin-assembly line is poorly known. However, bioinformatics and functional analyses have shown that ClbQ, a predicted type II thioesterase, could be involved in this process and therefore plays an important role in the control of the flux of colibactin production [42,44]. Once precolibactin synthesis is finished, the prodrug is taken in charge by ClbM, a multidrug and toxic compound extrusion (MATE) transporter, and released into the periplasmic space [45] (Figure 4). The crystal structure of ClbM has been determined [45] and revealed a large binding pocket allowing for the accommodation of large precolibactin molecules of 700–900 Da as previously reported (see Section 4. Colibactin Structure, below) [42,43,46]. Once into the periplasmic space, precolibactin is matured by the ClbP peptidase, which will generate the mature colibactin [47,48] via removal of the *N*-myristoyl-d-Asn side chain [34,35]. However, the mechanism by which colibactin is exported outside the bacteria is still unexplained. It is believed that activation of the prodrug into the periplasm is a strategy used by bacteria to protect their own DNA against the deleterious effect of colibactin. To prevent the side effects of any colibactin present in the cytoplasm, *pks E. coli* produce ClbS, which could sequester the colibactin and therefore protect DNA [49]. The 3D structure of ClbS was recently solved and revealed a potential colibactin binding site as well as similarities with hydrolases. Functional studies demonstrated that ClbS possesses cyclopropane hydrolase activity, which is able to convert genotoxic colibactin into an innocuous compound [50].

## 4. Colibactin Structure

**Figure 5:**
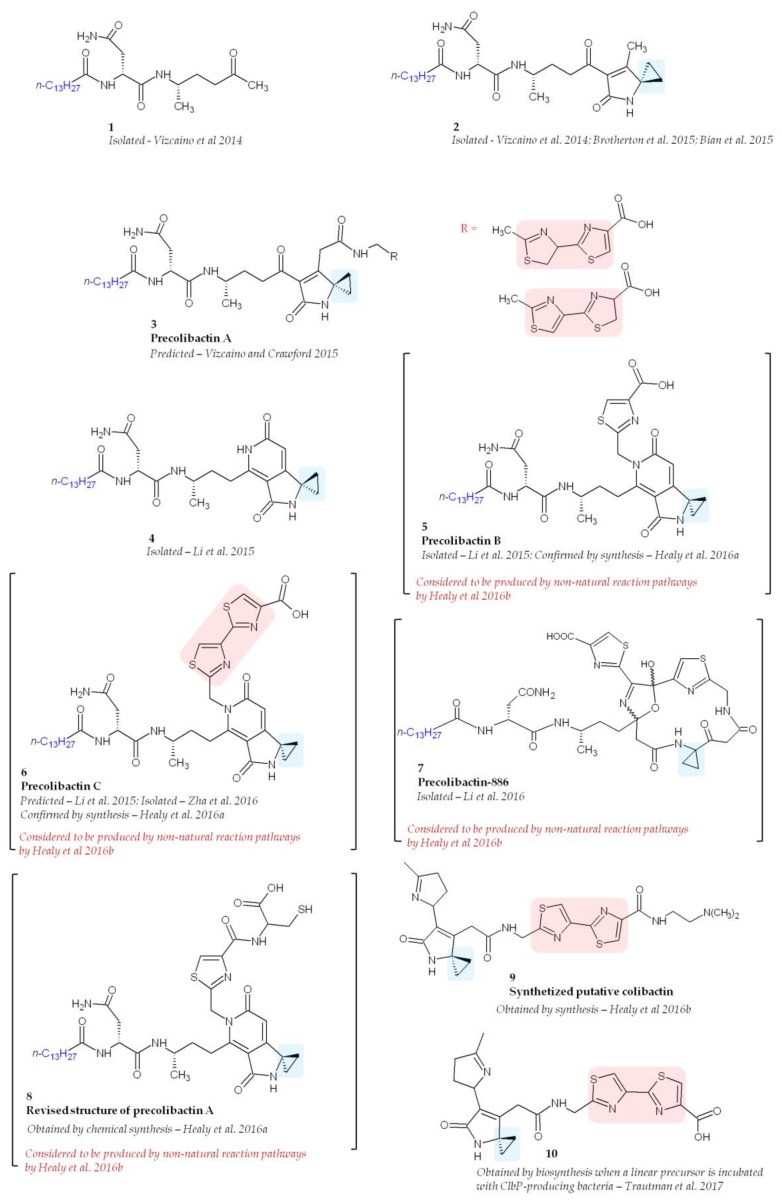
Structure of isolated, predicted, and synthetized precolibactins. Adapted from Reference [40]. Blue boxes represent cyclopropane structures responsible for DNA-alkylation. Red boxes represent bithiazole motifs which are likely able to intercalate DNA.

Despite intensive research, the structure of colibactin remains unknown probably owing to its high instability. Since colibactin cannot be purified directly from a *pks E. coli* culture media, a common employed strategy is to compare bacterial metabolites in *pks E. coli* and *pks E. coli* deficient for colibactin production (Δ*clb E. coli*). In Δ*clb E. coli*, precolibactins are accumulated, allowing their identification by mass spectrometry. However, the purification and characterization of precolibactins still remain a challenge, and compounds isolated so far have been obtained in extremely small quantities. Bian and colleagues were the first try to purify precolibactin molecules [34]. They isolated, from 48 L cultures of *E. coli*, 1.8 mg of *N*-myristoyl-d-Asn and a number of analogues with different acyl chain lengths (C_12_ to C_16_). However, because of the low yields, it was not possible to purify them for further elucidation and the purified product *N*-myristoyl-d-Asn had no cytotoxic activity. Vizcaino and colleagues also reported the presence of *N*-myristoyl-d-asparagine and several new colibactin metabolites [22] (Figure 5, molecule **1**). Unfortunately, none of these compounds exerted genotoxic activity against HeLa human epithelial cells, suggesting that other metabolites within the colibactin network possess this activity.

A molecule containing a spiro-cyclopropane, a structural motif rare among NRPS and PKS products, was reported in 2015 (Figure 5, molecule **2**) [43,51,52]. There is no homologue of known cyclopropane biosynthetic enzymes encoded in the *pks* island underlying the complexity of colibactin biosynthesis. Spiro-cyclopropane resembles the ring systems found in DNA-alkylation agents such as the duocarmycins [53] and illudins [54], suggesting that DNA alkylation could be important for the genotoxic activity of colibactin. Interestingly, this compound was shown in vitro to be able to crosslink DNA of linearized plasmids [43]. This activity was not observed when DNA-crosslinking experiments were performed using a precolibactin lacking the spirobicyclic motif, confirming the importance of this motif [43]. It has been recently shown that *pks E. coli* are able to induce DNA interstrand cross-links *in cellulo* [55]. In eukaryotic cells, DNA interstrand crosslinks induce multiple DNA repair machineries and the downstream DNA double-strand breaks [56]. Thus, it has been suggested that genotoxic activities of colibactin may account for DNA-crosslinking [43]. On the basis of the structure of this purified precolibactin, Vizcaino and Crawford proposed a more advanced compound that harbours a spirobicyclic structure and named it precolibactin A [43] (Figure 5, molecule **3**). Interestingly, using LC–MS, it was found that ClbP is able to process the precolibactin A metabolite, thereby confirming that the spirocyclic ring motif is present in precolibactin [52]. 

Large-scale cultures confirmed the presence of the compounds already described [43,52] and revealed new precolibactin molecules harbouring a thiazole ring (such as precolibactin B) (Figure 5, molecules **4**, and **5**) [46]. Compounds containing a thiazole, which is a heterocyclic ring that contains both sulphur and nitrogen, have been previously reported to be involved in DNA damage owing to the ability of thiazole to interact with DNA [57]. Using purified precolibactin B, Li and colleagues predicted the structure of a more advanced precolibactin containing a bithiazole motif (Figure 5, molecule **6**, precolibactin C) [46] which has been later purified [37]. The bithiazole motif is observed for example in bleomycin and is thought to intercalate into the DNA duplex [58].

The most advanced precolibactin compounds purified so far have been obtained by Li and co-workers starting from 1000 L of media [42]. To purify late-stage products, they used a Δ*clbQ* Δ*clbP* strain since ClbP is the enzyme responsible for final colibactin maturation [47,48] and ClbQ for the off-loading of precolibactin intermediates [42,44]. Using this strategy they were able to purify 2.8 mg of a 886 Da compound named precolibactin-886 [42] (Figure 5, molecule **7**). Interestingly, this compound exerted a greater cytotoxic effect on human epithelial cells (HCT-116 and HeLa) than less-advanced precolibactins already purified [42]. These studies were promising and suggested that we are close to solving for the structure of colibactin. However, this enthusiastic perspective has been tempered by studies conducted by Healy and colleagues, who suggested that metabolites produced by Δ*clbP* strains such as precolibactin-886 could arise from non-natural reaction pathways [40,59]. They focused on precolibactins A, B, and C and using chemical synthesis confirmed the structure of precolibactin B and C and proposed a revised model of precolibactin A (Figure 5, molecule **8**) [40]. However, the same authors, still using chemical synthesis, demonstrated later that precolibactins B and C and revised precolibactin A were probably not produced in vivo [59]. Finally, using a synthesis strategy instead of purification from bacterial culture, they proposed the structure of 13 synthetic colibactin derivatives and showed that cyclopropane is crucial for efficient DNA alkylation by colibactin (Figure 5, molecule **9** which had the highest DNA-alkylation properties in vitro) [59]. In order to be validated, these synthetized “colibactins” will have to be found in *pks E. coli*. Interestingly, a new reactive precolibactin metabolite structurally close to molecule **9** was characterized a few months ago from bacteria [39] (Figure 5, molecule **10**). Although this latest purified molecule contains potentially genotoxic structures, its genotoxic activity has not yet been tested.

## 5. Regulation of Colibactin Production

### 5.1. In Vitro Approaches

Given the serious side effects of colibactin on cells, it is important to understand the regulation of its synthesis. Since so far it has been impossible to assay colibactin concentration, the easiest way to assess the regulation of its expression is to measure the transcriptional levels of *clb* genes and to count in vitro the number of megalocytes (a direct consequence of colibactin-induced DNA breaks and cell cycle arrest) after infection. Bacterial culture conditions are important, in particular shaking since it increases the transcription levels of *clbA* to *clbH* genes [60]. The composition of the culture medium also seems to have an effect on colibactin production. It has been recently reported that iron-limiting conditions induce an increase in *clbA* transcription via pathways dependent and independent of the ferric uptake regulator Fur and the small noncoding RNA *RyhB* [61,62]. Finally, although its function is not fully understood, the *E. coli* heat shock protein Hsp90*_Ec_* has been shown to be crucial for the production of colibactin [63]. Authors have speculated that Hsp90*_Ec_* can either facilitate the folding of Clb proteins and/or increase their stability. 

### 5.2. In Vivo Approaches

To our knowledge, few data are available about the effects of in vivo conditions on colibactin expression. In *E. coli* strains isolated from urinary infections, it has been shown that the expression of the *pks* island was up-regulated when urine was used as the culture medium [64]. More importantly, a high expression level of *pks* genes was also observed in patients with urinary tract infection [64].

Two elegant studies using mouse models have assessed the regulation of colibactin synthesis in the gut. First, Arthur et al., investigated the effect of intestinal inflammation and colorectal cancer development using germ free *Il10*^-/-^ mice (colitis) and AOM/*Il10*^-/-^ mice (colorectal cancer) monocolonized with a *pks E. coli* strain isolated from a mouse gut [65]. Transcriptomic analysis showed that the expression of 66 *E. coli* genes was driven by cancer status. Interestingly, 5 *pks* island genes (*clbG*, *H*, *L*, *M,* and *S*) were upregulated among the 66 genes significantly affected. This important study clearly suggests that variations in the microenvironment during colorectal cancer development directly affect colibactin expression, pointing to the complexity of *pks* gene regulation. Further evidence of this complexity has been provided by an assessment of the genotoxicity of *pks E. coli* exposed to deoxynivalenol (DON), the most prevalent mycotoxin food contaminant. DON exacerbated the genotoxicity of *pks E. coli* in vitro and in rats intestinal epithelial cells [66]. DON alone did not exert any genotoxic effect and had no effect on bacterial growth, microbiota composition, or expression of *clb* genes. These observations suggest that synergy between toxins may occur. Although the regulation of colibactin synthesis and its possible interactions with other toxins/food contaminants remain largely unknown, these recent studies show that the environment plays a crucial role in the production and genotoxicity of colibactin. Thus, we can speculate that many people harbour *pks* bacteria in which colibactin expression is repressed. However, when certain changes occur in external or internal conditions, colibactin expression can be turned on and consequently profoundly affect health.

## 6. Roles of Colibactin

The signature of colibactin is the induction of DNA double-strand breaks, in vitro and in vivo, [2,3,8,14,67] and chromosome aberrations [3]. The genotoxic activity of *pks E. coli* requires live bacteria and direct contacts with epithelial cells, suggesting that colibactin is either poorly diffusible or highly unstable [2,68]. Thus, it has been hypothesized that colibactin-producing bacteria could profoundly affect their host’s health.

### 6.1. Colibactin and Gut Homeostasis

Since clinical studies showed that the newborn’s gut is frequently colonized by *pks E. coli* [7,8], experimental studies have been conducted to estimate the effect of early intestinal tract colonization by these strains. The natural transmission of *pks E. coli* strains to the offspring has been confirmed in rats [8]. This mother to offspring transmission profoundly alters gut physiology, and the alterations are transmissible across generations [8]. The presence of *pks E. coli* in newborn rats impaired intestinal permeability [8,69], which enhanced the trans-epithelial passage of luminal antigens and lead to increased immune function [69]. This profound modification of immune response contributes to the defect of oral tolerance and could facilitate the development of dysregulated immune-mediated diseases. In addition, Payros and colleagues observed that maternally acquired commensal genotoxic *E. coli* strains exacerbate DNA damage in intestinal epithelial cells of neonates and cause chromosomal instability [8]. Furthermore, the physiology of epithelial cells is completely modified by acquired *pks E. coli* since the bacteria increase the rate of cell apoptosis, which is compensated by an increase in cell proliferation leading to an abnormal crypt fission rate [8]. Although no cases of spontaneous colorectal cancer (CRC) have been reported, we can speculate that the alteration in gut renewal could favour the development of cancer if intestinal epithelial cells are in contact with carcinogen compounds or high levels of inflammatory molecules.

### 6.2. Colibactin and Colorectal Cancer

Because *pks E. coli* are frequently found in gut microbiota [4,5,6] and DNA alterations are associated with cancers, numerous studies have been performed to understand the potential role of these bacteria in CRC.

Most CRCs (~90%) are sporadic and therefore influenced by external factors such as diet and microbiota [70,71,72,73]. *E. coli* is highly suspected of being involved in CRC development. It has been known for almost 20 years that human CRC biopsies are highly colonized by *E. coli* [74]. Closer observation of these bacteria show that they are able to produce several cyclomodulins and in particular colibactin [12,14,15]. Results showed that colibactin-producing *E. coli* are found in 55–67% of colorectal cancer patients but in less than 20% of controls [12,14]. In a recent study using PCR, *clbA* was found to be over-represented in the stools of CRC patients compared to those of controls (56.4% vs. 18.5%, respectively) [13]. Patients with familial adenomatous polyposis (FAP) develop benign precursor lesions (polyps) early in life due to germline mutation in the *APC* (adenomatous polyposis coli) tumour suppressor gene [75]. It has been reported that mucosa of FAP patients was significantly associated with *pks E. coli* (68%) compare to healthy subject mucosa (22%) [76]. All these studies were performed in Europe (UK [14,15], France [12], and Sweden [13]) and in the USA [76]. To date, only one study, performed in Japan, has not reported any difference in the prevalence of *pks* positive genes found in the faeces of CRC patients and healthy controls [77]. In this study 43% of CRC patients were positive for *pks* (which is consistent with previous reports). In contrast, 46% of controls were positive for *pks*, which is much higher than the prevalence reported in previous studies. Microbiota composition depends on the geographic area considered [78], which could explain why the distribution of the *pks* island in the Japanese sample was different than that in Europe and in the USA. It would be interesting to perform more epidemiological studies in Asia to see if this discrepancy is confirmed.

A bacterial driver-passenger model has recently been proposed in the development of CRC [79]. In this model, some bacteria (termed “*bacterial drivers*”) would initiate the CRC thereby altering the intestinal niche that favours the proliferation of opportunistic bacteria (termed “*bacterial passengers*”). Current experimental data on animals suggest that *pks E. coli* are unable to spontaneously induce CRC [8]. However, in mice predisposed to CRC, the same bacteria increase the severity of the disease [14,16,17,18]. This potentially suggests that they have a passenger role, but given the deleterious effect on DNA caused by colibactin, a driver role is also feasible. To induce DNA damage and play a driver role, *pks* bacteria need to be in close contact with intestinal stem cells [2] and to be exposed to favourable environmental conditions (in order to produce colibactin). Thus, although the Japanese patients had a high carriage of *pks E. coli*, until their exposure to the specific conditions required for the production of colibactin and for the contact between bacteria and epithelial cells, *pks E. coli* will not have deleterious effects on intestinal epithelial cells.

Colibactin-producing *E. coli* increased epithelial cell proliferation in vivo [8,16,28], tumour invasion, and the number of tumours in diverse CRC mouse models [14,16,17,18]. This suggests that, on a broader level, they could promote human CRC development. In addition, colibactin-producing *E. coli* induced inhibition of the mutL homologue 1 (MLH1) mismatch repair protein [80], which could lead to the genomic instability associated with *pks E. coli* infection [3,8]. However, colibactin always induces cell cycle arrest, which is in contradiction with the promotion of cancer. One suggested explanation of these conflicting observations is that cycle arrest coincides with a cellular senescence in various cell types including intestinal epithelial cells [17,81]. It has been observed that *pks E. coli* induces the expression of microRNA-20a-5p, which is responsible for p53 post-translational modification (accumulation of small ubiquitin-like modifier (SUMO)-conjugated p53), which in turn leads to cellular senescence [17,82]. However, senescence is also observed in p53 mutated cells, which is suggestive of a cellular bypass process. Senescent intestinal epithelial cells are still metabolically active and produce a senescence-associated phenotype that stimulates the proliferation of uninfected cells both in vitro and in vivo [17,82]. Importantly, those pathways leading to cellular senescence were also found to be dysregulated in human CRC biopsies colonized by *pks E. coli* [17]. All of these data strongly support the hypothesis that colibactin plays a role in CRC. Thus, inhibition of crucial proteins (Clbs) involved in colibactin synthesis has been proposed [45,67], and inhibitors targeting ClbP have been developed [67]. Interestingly, these molecules were able to abolish colibactin-induced DNA damage both in vitro and in a CRC mouse model, and as a result, decreased the number of tumours [67,83]. This proof of concept suggests that personalized medicine in the future should consider not only the genetics of the tumour but also the tumour-associated bacteria. In the treatment of CRC patients colonized by *pks* bacteria, the use of inhibitors of colibactin synthesis could be an interesting adjuvant strategy to limit cell proliferation [83].

### 6.3. Colibactin and Neonatal Sepsis/Meningitis

As *pks* is widely distributed among *E. coli* K1 strains isolated from cases of meningitis [10], its function has been investigated in the aetiology of the disease. First, the *pks* island is functional in *E. coli* K1 strains and induces DNA damage but, more importantly, *pks* contributes to the bacterial virulence [10,84]. In newborns, systemic infection by *E. coli* K1 necessarily involves the translocation of bacteria from the immature gut to the blood. Using animals models, it has been shown that inactivation of *clbA* or *clbP* reduces the capacity of bacteria to colonize the gut and consequently to translocate to the blood [10,69]. Once in the blood stream, *pks E. coli* induce apoptosis of T lymphocytes and exacerbate lymphopenia associated with septicaemia, thereby decreasing the survival rate of rats with sepsis [84]. Interestingly, a study made in Taiwan reported that abrogation of colibactin production in *Klebsiella pneumoniae* (the most common pathogen of community-acquired meningitis in Taiwan) significantly decreased *K. pneumoniae* hyper-virulence in the key steps towards the development of meningitis [85].

### 6.4. Other Roles Played by Colibactin

While numerous studies have evidenced the deleterious effects of colibactin, some studies have pointed out that things are probably more complicated than initially thought. For example, *E. coli* Nissle 1917, a probiotic strain commercially available and used to treat intestinal disorders [86], possesses the *pks* island [20]. A functional *pks* island is mandatory for this strain to exercise its anti-inflammatory effects in a colitis mouse model [20], suggesting that one or more anti-inflammatory compounds are synthetized by the *pks* island. In addition, the *pks* island is responsible, in this probiotic strain, for the production of an analgesic lipopeptid (C12AsnGABA*OH*), which is able to cross the epithelial barrier and inhibit the calcium flux induced by nociceptor activation in sensory neurons via the GABA_B_ receptor. As a result, this abrogates visceral hypersensitivity in mice [23]. Another interesting function of the *pks* island is the production of one or more antibiotic molecules. The *N*-myristoyl-d-Asn released during colibactin maturation exerts mild growth-inhibitory activity against *Bacillus subtilis* NCIB 3610 [22]. On the other hand, not yet purified compound(s) are able to inhibit the growth of *Staphylococcus aureus* and, more importantly, that of methicillin-resistant *S. aureus* strains and strains resistant to “last-resort” antistaphylococcal antibiotics [21]. These interesting findings highlight the need to better understand the chemical steps involved in colibactin synthesis, in order to be able to isolate and/or synthetize compounds of therapeutic interest.

## 7. Conclusions

In just one decade, *pks* bacteria have emerged as major players in human health. Owing to its pleiotropic effects, colibactin profoundly influences cellular physiology, inducing DNA breaks that lead to senescence or apoptosis. Numerous in vitro and, more importantly, in vivo studies have evidenced the harmful effects of colibactin in sepsis, meningitis, and the intestinal tract. Colibactin is also strongly suspected of being involved in the development of CRC. The use of Clbs inhibitors has been suggested as a means of preventing its deleterious effects and a proof of concept has been made. However, in addition to the genotoxic effects of colibactin, studies have clearly shown that some products generated by the *pks* island are of interest since they exert antibiotic, analgesic, or anti-inflammatory effects. Further studies are needed to find a means of preventing the genotoxic effect of colibactin, and work should be done on the purification of the diverse products generated by the *pks* island, since some of them could be potentially used in human medicine.

## References

[1] Nougayrède J.-P., Taieb F., De Rycke J., Oswald E. (2005). Cyclomodulins: bacterial effectors that modulate the eukaryotic cell cycle. Trends Microbiol..

[2] Nougayrède J.-P., Homburg S., Taieb F., Boury M., Brzuszkiewicz E., Gottschalk G., Buchrieser C., Hacker J., Dobrindt U., Oswald E. (2006). Escherichia coli induces DNA double-strand breaks in eukaryotic cells. Science.

[3] Cuevas-Ramos G., Petit C.R., Marcq I., Boury M., Oswald E., Nougayrède J.-P. (2010). Escherichia coli induces DNA damage in vivo and triggers genomic instability in mammalian cells. Proc. Natl. Acad. Sci. USA.

[4] Dubois D., Delmas J., Cady A., Robin F., Sivignon A., Oswald E., Bonnet R. (2010). Cyclomodulins in urosepsis strains of Escherichia coli. J. Clin. Microbiol..

[5] Johnson J.R., Johnston B., Kuskowski M.A., Nougayrede J.-P., Oswald E. (2008). Molecular epidemiology and phylogenetic distribution of the Escherichia coli pks genomic island. J. Clin. Microbiol..

[6] Putze J., Hennequin C., Nougayrède J.-P., Zhang W., Homburg S., Karch H., Bringer M.-A., Fayolle C., Carniel E., Rabsch W. (2009). Genetic structure and distribution of the colibactin genomic island among members of the family Enterobacteriaceae. Infect. Immun..

[7] Nowrouzian F.L., Oswald E. (2012). Escherichia coli strains with the capacity for long-term persistence in the bowel microbiota carry the potentially genotoxic pks island. Microb. Pathog..

[8] Payros D., Secher T., Boury M., Brehin C., Ménard S., Salvador-Cartier C., Cuevas-Ramos G., Watrin C., Marcq I., Nougayrède J.-P. (2014). Maternally acquired genotoxic Escherichia coli alters offspring’s intestinal homeostasis. Gut Microbes.

[9] Micenková L., Beňová A., Frankovičová L., Bosák J., Vrba M., Ševčíková A., Kmeťová M., Šmajs D. (2017). Human Escherichia coli isolates from hemocultures: Septicemia linked to urogenital tract infections is caused by isolates harboring more virulence genes than bacteraemia linked to other conditions. Int. J. Med. Microbiol..

[10] McCarthy A.J., Martin P., Cloup E., Stabler R.A., Oswald E., Taylor P.W. (2015). The Genotoxin Colibactin Is a Determinant of Virulence in Escherichia coli K1 Experimental Neonatal Systemic Infection. Infect. Immun..

[11] Krieger J.N., Dobrindt U., Riley D.E., Oswald E. (2011). Acute Escherichia coli prostatitis in previously health young men: bacterial virulence factors, antimicrobial resistance, and clinical outcomes. Urology.

[12] Buc E., Dubois D., Sauvanet P., Raisch J., Delmas J., Darfeuille-Michaud A., Pezet D., Bonnet R. (2013). High prevalence of mucosa-associated E. coli producing cyclomodulin and genotoxin in colon cancer. PLoS ONE.

[13] Eklöf V., Löfgren-Burström A., Zingmark C., Edin S., Larsson P., Karling P., Alexeyev O., Rutegård J., Wikberg M.L., Palmqvist R. (2017). Cancer-associated fecal microbial markers in colorectal cancer detection. Int. J. Cancer.

[14] Arthur J.C., Perez-Chanona E., Mühlbauer M., Tomkovich S., Uronis J.M., Fan T.-J., Campbell B.J., Abujamel T., Dogan B., Rogers A.B. (2012). Intestinal inflammation targets cancer-inducing activity of the microbiota. Science.

[15] Prorok-Hamon M., Friswell M.K., Alswied A., Roberts C.L., Song F., Flanagan P.K., Knight P., Codling C., Marchesi J.R., Winstanley C. (2014). Colonic mucosa-associated diffusely adherent afaC+ Escherichia coli expressing lpfA and pks are increased in inflammatory bowel disease and colon cancer. Gut.

[16] Bonnet M., Buc E., Sauvanet P., Darcha C., Dubois D., Pereira B., Déchelotte P., Bonnet R., Pezet D., Darfeuille-Michaud A. (2014). Colonization of the human gut by E. coli and colorectal cancer risk. Clin. Cancer Res..

[17] Cougnoux A., Dalmasso G., Martinez R., Buc E., Delmas J., Gibold L., Sauvanet P., Darcha C., Déchelotte P., Bonnet M. (2014). Bacterial genotoxin colibactin promotes colon tumour growth by inducing a senescence-associated secretory phenotype. Gut.

[18] Tomkovich S., Yang Y., Winglee K., Gauthier J., Mühlbauer M., Sun X., Mohamadzadeh M., Liu X., Martin P., Wang G.P. (2017). Locoregional Effects of Microbiota in a Preclinical Model of Colon Carcinogenesis. Cancer Res..

[19] Fischbach M.A., Walsh C.T. (2006). Assembly-line enzymology for polyketide and nonribosomal Peptide antibiotics: logic, machinery, and mechanisms. Chem. Rev..

[20] Olier M., Marcq I., Salvador-Cartier C., Secher T., Dobrindt U., Boury M., Bacquié V., Pénary M., Gaultier E., Nougayrède J.-P. (2012). Genotoxicity of Escherichia coli Nissle 1917 strain cannot be dissociated from its probiotic activity. Gut Microbes.

[21] Faïs T., Cougnoux A., Dalmasso G., Laurent F., Delmas J., Bonnet R. (2016). Antibiotic Activity of Escherichia coli against Multiresistant Staphylococcus aureus. Antimicrob. Agents Chemother..

[22] Vizcaino M.I., Engel P., Trautman E., Crawford J.M. (2014). Comparative metabolomics and structural characterizations illuminate colibactin pathway-dependent small molecules. J. Am. Chem. Soc..

[23] Pérez-Berezo T., Pujo J., Martin P., Le Faouder P., Galano J.-M., Guy A., Knauf C., Tabet J.C., Tronnet S., Barreau F. (2017). Identification of an analgesic lipopeptide produced by the probiotic Escherichia coli strain Nissle 1917. Nat. Commun..

[24] Flores-Mireles A.L., Walker J.N., Caparon M., Hultgren S.J. (2015). Urinary tract infections: epidemiology, mechanisms of infection and treatment options. Nat. Rev. Microbiol..

[25] Sarff L.D., McCracken G.H., Schiffer M.S., Glode M.P., Robbins J.B., Orskov I., Orskov F. (1975). Epidemiology of Escherichia coli K1 in healthy and diseased newborns. Lancet.

[26] Clermont O., Christenson J.K., Denamur E., Gordon D.M. (2013). The Clermont Escherichia coli phylo-typing method revisited: improvement of specificity and detection of new phylo-groups. Environ. Microbiol. Rep..

[27] Escobar-Páramo P., Clermont O., Blanc-Potard A.-B., Bui H., Le Bouguénec C., Denamur E. (2004). A specific genetic background is required for acquisition and expression of virulence factors in Escherichia coli. Mol. Biol. Evol..

[28] Raisch J., Buc E., Bonnet M., Sauvanet P., Vazeille E., de Vallée A., Déchelotte P., Darcha C., Pezet D., Bonnet R. (2014). Colon cancer-associated B2 Escherichia coli colonize gut mucosa and promote cell proliferation. World J. Gastroenterol..

[29] Tenaillon O., Skurnik D., Picard B., Denamur E. (2010). The population genetics of commensal Escherichia coli. Nat. Rev. Microbiol..

[30] Lai Y.-C., Lin A.-C., Chiang M.-K., Dai Y.-H., Hsu C.-C., Lu M.-C., Liau C.-Y., Chen Y.-T. (2014). Genotoxic Klebsiella pneumoniae in Taiwan. PLoS ONE.

[31] Fung C.P., Hu B.S., Chang F.Y., Lee S.C., Kuo B.I., Ho M., Siu L.K., Liu C.Y. (2000). A 5-year study of the seroepidemiology of Klebsiella pneumoniae: high prevalence of capsular serotype K1 in Taiwan and implication for vaccine efficacy. J. Infect. Dis..

[32] Thompson W., Romance L., Bialkowska-Hobrazanska H., Rennie R.P., Ashton F., Nicolle L.E. (1993). Klebsiella pneumoniae infection on a rehabilitation unit: comparison of epidemiologic typing methods. Infect. Control Hosp. Epidemiol..

[33] Chen Y.-T., Lai Y.-C., Tan M.-C., Hsieh L.-Y., Wang J.-T., Shiau Y.-R., Wang H.-Y., Lin A.-C., Lai J.-F., Huang I.-W. (2017). Prevalence and characteristics of pks genotoxin gene cluster-positive clinical Klebsiella pneumoniae isolates in Taiwan. Sci. Rep..

[34] Bian X., Fu J., Plaza A., Herrmann J., Pistorius D., Stewart A.F., Zhang Y., Müller R. (2013). In vivo evidence for a prodrug activation mechanism during colibactin maturation. Chembiochem.

[35] Brotherton C.A., Balskus E.P. (2013). A prodrug resistance mechanism is involved in colibactin biosynthesis and cytotoxicity. J. Am. Chem. Soc..

[36] Martin P., Marcq I., Magistro G., Penary M., Garcie C., Payros D., Boury M., Olier M., Nougayrède J.-P., Audebert M. (2013). Interplay between siderophores and colibactin genotoxin biosynthetic pathways in Escherichia coli. PLoS Pathog..

[37] Zha L., Wilson M.R., Brotherton C.A., Balskus E.P. (2016). Characterization of Polyketide Synthase Machinery from the pks Island Facilitates Isolation of a Candidate Precolibactin. ACS Chem. Biol..

[38] Zha L., Jiang Y., Henke M.T., Wilson M.R., Wang J.X., Kelleher N.L., Balskus E.P. (2017). Colibactin assembly line enzymes use S-adenosylmethionine to build a cyclopropane ring. Nat. Chem. Biol..

[39] Trautman E.P., Healy A.R., Shine E.E., Herzon S.B., Crawford J.M. (2017). Domain-Targeted Metabolomics Delineates the Heterocycle Assembly Steps of Colibactin Biosynthesis. J. Am. Chem. Soc..

[40] Healy A.R., Vizcaino M.I., Crawford J.M., Herzon S.B. (2016). Convergent and Modular Synthesis of Candidate Precolibactins. Structural Revision of Precolibactin A. J. Am. Chem. Soc..

[41] Brachmann A.O., Garcie C., Wu V., Martin P., Ueoka R., Oswald E., Piel J. (2015). Colibactin biosynthesis and biological activity depend on the rare aminomalonyl polyketide precursor. Chem. Commun. (Camb.).

[42] Li Z.-R., Li J., Gu J.-P., Lai J.Y.H., Duggan B.M., Zhang W.-P., Li Z.-L., Li Y.-X., Tong R.-B., Xu Y. (2016). Divergent biosynthesis yields a cytotoxic aminomalonate-containing precolibactin. Nat. Chem. Biol..

[43] Vizcaino M.I., Crawford J.M. (2015). The colibactin warhead crosslinks DNA. Nat. Chem..

[44] Guntaka N.S., Healy A.R., Crawford J.M., Herzon S.B., Bruner S.D. (2017). Structure and Functional Analysis of ClbQ, an Unusual Intermediate-Releasing Thioesterase from the Colibactin Biosynthetic Pathway. ACS Chem. Biol..

[45] Mousa J.J., Yang Y., Tomkovich S., Shima A., Newsome R.C., Tripathi P., Oswald E., Bruner S.D., Jobin C. (2016). MATE transport of the E. coli-derived genotoxin colibactin. Nat. Microbiol..

[46] Li Z.-R., Li Y., Lai J.Y.H., Tang J., Wang B., Lu L., Zhu G., Wu X., Xu Y., Qian P.-Y. (2015). Critical Intermediates Reveal New Biosynthetic Events in the Enigmatic Colibactin Pathway. Chembiochem.

[47] Cougnoux A., Gibold L., Robin F., Dubois D., Pradel N., Darfeuille-Michaud A., Dalmasso G., Delmas J., Bonnet R. (2012). Analysis of structure-function relationships in the colibactin-maturating enzyme ClbP. J. Mol. Biol..

[48] Dubois D., Baron O., Cougnoux A., Delmas J., Pradel N., Boury M., Bouchon B., Bringer M.-A., Nougayrède J.-P., Oswald E., Bonnet R. (2011). ClbP is a prototype of a peptidase subgroup involved in biosynthesis of nonribosomal peptides. J. Biol. Chem..

[49] Bossuet-Greif N., Dubois D., Petit C., Tronnet S., Martin P., Bonnet R., Oswald E., Nougayrède J.-P. (2016). Escherichia coli ClbS is a colibactin resistance protein. Mol. Microbiol..

[50] Tripathi P., Shine E.E., Healy A.R., Kim C.S., Herzon S.B., Bruner S.D., Crawford J.M. (2017). ClbS is a cyclopropane hydrolase that confers colibactin resistance. J. Am. Chem. Soc..

[51] Bian X., Plaza A., Zhang Y., Müller R. (2015). Two more pieces of the colibactin genotoxin puzzle from Escherichia coli show incorporation of an unusual 1-aminocyclopropanecarboxylic acid moiety. Chem. Sci..

[52] Brotherton C.A., Wilson M., Byrd G., Balskus E.P. (2015). Isolation of a metabolite from the pks island provides insights into colibactin biosynthesis and activity. Org. Lett..

[53] Boger D.L., Johnson D.S. (1995). CC-1065 and the duocarmycins: unraveling the keys to a new class of naturally derived DNA alkylating agents. Proc. Natl. Acad. Sci. USA.

[54] Tanasova M., Sturla S.J. (2012). Chemistry and biology of acylfulvenes: sesquiterpene-derived antitumor agents. Chem. Rev..

[55] Bossuet-Greif N., Vignard J., Taieb F., Mirey G., Dubois D., Petit C., Oswald E., Nougayrède J.-P. (2018). The Colibactin Genotoxin Generates DNA Interstrand Cross-Links in Infected Cells. MBio.

[56] Deans A.J., West S.C. (2011). DNA interstrand crosslink repair and cancer. Nat. Rev. Cancer.

[57] Roy R.S., Gehring A.M., Milne J.C., Belshaw P.J., Walsh C.T. (1999). Thiazole and oxazole peptides: biosynthesis and molecular machinery. Nat. Prod. Rep..

[58] Povirk L.F., Hogan M., Dattagupta N. (1979). Binding of bleomycin to DNA: intercalation of the bithiazole rings. Biochemistry.

[59] Healy A.R., Nikolayevskiy H., Patel J.R., Crawford J.M., Herzon S.B. (2016). A Mechanistic Model for Colibactin-Induced Genotoxicity. J. Am. Chem. Soc..

[60] Homburg S., Oswald E., Hacker J., Dobrindt U. (2007). Expression analysis of the colibactin gene cluster coding for a novel polyketide in Escherichia coli. FEMS Microbiol. Lett..

[61] Tronnet S., Garcie C., Rehm N., Dobrindt U., Oswald E., Martin P. (2016). Iron Homeostasis Regulates the Genotoxicity of Escherichia coli That Produces Colibactin. Infect. Immun..

[62] Tronnet S., Garcie C., Brachmann A.O., Piel J., Oswald E., Martin P. (2017). High iron supply inhibits the synthesis of the genotoxin colibactin by pathogenic Escherichia coli through a non-canonical Fur/RyhB-mediated pathway. Pathog. Dis..

[63] Garcie C., Tronnet S., Garénaux A., McCarthy A.J., Brachmann A.O., Pénary M., Houle S., Nougayrède J.-P., Piel J., Taylor P.W. (2016). The Bacterial Stress-Responsive Hsp90 Chaperone (HtpG) Is Required for the Production of the Genotoxin Colibactin and the Siderophore Yersiniabactin in Escherichia coli. J. Infect. Dis..

[64] Hancock V., Seshasayee A.S., Ussery D.W., Luscombe N.M., Klemm P. (2008). Transcriptomics and adaptive genomics of the asymptomatic bacteriuria Escherichia coli strain 83972. Mol. Genet. Genom..

[65] Arthur J.C., Gharaibeh R.Z., Mühlbauer M., Perez-Chanona E., Uronis J.M., McCafferty J., Fodor A.A., Jobin C. (2014). Microbial genomic analysis reveals the essential role of inflammation in bacteria-induced colorectal cancer. Nat. Commun..

[66] Payros D., Dobrindt U., Martin P., Secher T., Bracarense A.P.F.L., Boury M., Laffitte J., Pinton P., Oswald E., Oswald I.P. (2017). The Food Contaminant Deoxynivalenol Exacerbates the Genotoxicity of Gut Microbiota. MBio.

[67] Cougnoux A., Delmas J., Gibold L., Faïs T., Romagnoli C., Robin F., Cuevas-Ramos G., Oswald E., Darfeuille-Michaud A., Prati F. (2016). Small-molecule inhibitors prevent the genotoxic and protumoural effects induced by colibactin-producing bacteria. Gut.

[68] Reuter C., Alzheimer M., Walles H., Oelschlaeger T.A. (2017). An Adherent Mucus Layer Attenuates the Genotoxic Effect of Colibactin. Cell. Microbiol..

[69] Secher T., Payros D., Brehin C., Boury M., Watrin C., Gillet M., Bernard-Cadenat I., Menard S., Theodorou V., Saoudi A. (2015). Oral tolerance failure upon neonatal gut colonization with Escherichia coli producing the genotoxin colibactin. Infect. Immun..

[70] Gagnière J., Raisch J., Veziant J., Barnich N., Bonnet R., Buc E., Bringer M.-A., Pezet D., Bonnet M. (2016). Gut microbiota imbalance and colorectal cancer. World J. Gastroenterol..

[71] Lucas C., Barnich N., Nguyen H.T.T. (2017). Microbiota, Inflammation and Colorectal Cancer. Int. J. Mol. Sci..

[72] Raisch J., Dalmasso G., Bonnet R., Barnich N., Bonnet M., Bringer M.-A. (2016). [How some commensal bacteria would exacerbate colorectal carcinogenesis?]. Med. Sci. (Paris).

[73] Weitz J., Koch M., Debus J., Höhler T., Galle P.R., Büchler M.W. (2005). Colorectal cancer. Lancet.

[74] Swidsinski A., Khilkin M., Kerjaschki D., Schreiber S., Ortner M., Weber J., Lochs H. (1998). Association between intraepithelial Escherichia coli and colorectal cancer. Gastroenterology.

[75] Kinzler K.W., Nilbert M.C., Su L.K., Vogelstein B., Bryan T.M., Levy D.B., Smith K.J., Preisinger A.C., Hedge P., McKechnie D. (1991). Identification of FAP locus genes from chromosome 5q21. Science.

[76] Dejea C.M., Fathi P., Craig J.M., Boleij A., Taddese R., Geis A.L., Wu X., DeStefano Shields C.E., Hechenbleikner E.M., Huso D.L. (2018). Patients with familial adenomatous polyposis harbor colonic biofilms containing tumorigenic bacteria. Science.

[77] Shimpoh T., Hirata Y., Ihara S., Suzuki N., Kinoshita H., Hayakawa Y., Ota Y., Narita A., Yoshida S., Yamada A. (2017). Prevalence of pks-positive Escherichia coli in Japanese patients with or without colorectal cancer. Gut Pathog..

[78] Lozupone C.A., Stombaugh J.I., Gordon J.I., Jansson J.K., Knight R. (2012). Diversity, stability and resilience of the human gut microbiota. Nature.

[79] Tjalsma H., Boleij A., Marchesi J.R., Dutilh B.E. (2012). A bacterial driver-passenger model for colorectal cancer: beyond the usual suspects. Nat. Rev. Microbiol..

[80] Gagnière J., Bonnin V., Jarrousse A.-S., Cardamone E., Agus A., Uhrhammer N., Sauvanet P., Déchelotte P., Barnich N., Bonnet R. (2017). Interactions between microsatellite instability and human gut colonization by Escherichia coli in colorectal cancer. Clin. Sci..

[81] Secher T., Samba-Louaka A., Oswald E., Nougayrède J.-P. (2013). Escherichia coli producing colibactin triggers premature and transmissible senescence in mammalian cells. PLoS ONE.

[82] Dalmasso G., Cougnoux A., Delmas J., Darfeuille-Michaud A., Bonnet R. (2014). The bacterial genotoxin colibactin promotes colon tumor growth by modifying the tumor microenvironment. Gut Microbes.

[83] Faïs T., Delmas J., Cougnoux A., Dalmasso G., Bonnet R. (2016). Targeting colorectal cancer-associated bacteria: A new area of research for personalized treatments. Gut Microbes.

[84] Marcq I., Martin P., Payros D., Cuevas-Ramos G., Boury M., Watrin C., Nougayrède J.-P., Olier M., Oswald E. (2014). The genotoxin colibactin exacerbates lymphopenia and decreases survival rate in mice infected with septicemic Escherichia coli. J. Infect. Dis..

[85] Lu M.-C., Chen Y.-T., Chiang M.-K., Wang Y.-C., Hsiao P.-Y., Huang Y.-J., Lin C.-T., Cheng C.-C., Liang C.-L., Lai Y.-C. (2017). Colibactin Contributes to the Hypervirulence of pks(+) K1 CC23 Klebsiella pneumoniae in Mouse Meningitis Infections. Front. Cell. Infect. Microbiol..

[86] Wassenaar T.M. (2016). Insights from 100 Years of Research with Probiotic *E. coli*. Eur. J. Microbiol. Immunol. (Bp).

